# Risk Factors Associated With Postoperative Complications Following Endovenous Laser Ablation for Varicose Veins

**DOI:** 10.1155/ijvm/8875397

**Published:** 2025-10-28

**Authors:** Aram Baram, Raz Kamaran Hama Ali, Chya Nasir Qadir, Shiba Ahmad Faqe

**Affiliations:** Branch of Clinical Sciences, College of Medicine, University of Sulaimani, Sulaymaniyah, Iraq

**Keywords:** 1470-nm laser, CEAP, complications, DVT, EHIT, EVLA, EVLT, VCSS

## Abstract

**Background:**

Endovenous laser ablation (EVLA) is a widely accepted treatment for varicose veins, offering effective results with minimal invasiveness. However, postoperative complications remain a concern. This study examines risk factors associated with adverse outcomes and evaluates long-term clinical efficacy over 48 months.

**Methods:**

This retrospective, single-center study was conducted from January 2020 to January 2024 and included 500 patients with symptomatic varicose veins due to saphenous vein incompetence. All underwent preoperative clinical and duplex ultrasound evaluation; Clinical, Etiological, Anatomical, and Pathophysiological (CEAP) classification; and Venous Clinical Severity Score (VCSS) scoring. Patients with postthrombotic syndrome, congenital malformations, or active DVT were excluded. EVLA using a 1470-nm laser was performed by an experienced vascular surgeon. Follow-up visits were scheduled periodically over 48 months. Primary outcomes included postoperative complications, while secondary outcomes focused on VCSS trends and patient satisfaction.

**Results:**

The cohort had a mean age of 38 years and was 56.8% female. Most were CEAP C4 or C5 with Grade 3 venous reflux. Minor complications included hematoma (6.4%), swelling (7.2%), infection (4%), and nerve injury (3.6%). Deep vein thrombosis occurred in 2% of patients; no pulmonary embolism or major cardiac events were reported. Endothermal heat–induced thrombosis occurred in 9%, with only 0.8% reaching Grade 3. Hypertension, diabetes, and obesity were associated with higher complication rates. By 48 months, all patients showed clinical improvement with VCSSs below 5.

**Conclusions:**

EVLA is a safe, effective treatment for varicose veins, even in advanced CEAP stages. Identifying patient-specific risk factors may help reduce complications and improve outcomes.

## 1. Introduction

Varicose veins (VVs) are the commonest manifestation of chronic venous disease (CVD), characterized by dilated and tortuous subcutaneous veins measuring at least 3 mm in diameter, in the standing position. These veins most commonly affect the lower limbs and exhibit a highly variable global prevalence, reported between 1% and 73% in women and 2% and 56% in men [1, 2].

The development of VVs is multifactorial, with risk factors such as body mass index (BMI), marital status, and educational background playing contributory roles [2]. Clinically, patients often present with symptoms including leg heaviness, swelling, itching, and fatigue. In more advanced stages, skin changes such as hyperpigmentation, eczema, lipodermatosclerosis, and venous ulcers may occur. Though often considered benign, VVs can lead to substantial structural and functional impairments in the venous system [3].

The CEAP (Clinical, Etiological, Anatomical, and Pathophysiological) classification system provides a standardized approach for assessing the severity of venous disease and guiding treatment decisions [1]. Management is tailored based on disease severity, patient preferences, potential risks, and cost-effectiveness [2]. In asymptomatic individuals, conservative treatment remains the first-line approach, including lifestyle changes, pharmacotherapy, and compression therapy. Among these, graduated compression stockings are often preferred for comfort and ease of use [1–3].

For symptomatic patients, venoactive drugs may improve venous tone and reduce capillary permeability [2]. Minimally invasive endovenous options, such as endovenous laser ablation (EVLA) and radiofrequency ablation (RFA), and steam ablation, are increasingly used. Nonthermal alternatives like MOCA and cyanoacrylate closure also offer effective options for selected cases [3, 4].

Recent studies support the safety and efficacy of endothermal ablation (ETA) techniques, showing comparable or better outcomes than traditional high ligation and stripping (HL/S). Clinical trials report favorable results for both RFA and EVLA regarding technical success, recurrence, and reintervention rates [5]. ETA is also associated with faster recovery, less postoperative discomfort, and improved quality of life [6, 7]. While HL/S was once the gold standard, current guidelines recommend it only when less invasive methods like EVLA, RFA, or sclerotherapy are unsuitable [5–7].

Despite its benefits, endovenous laser treatment (EVLT) is not without risks. Complications may include vein wall perforation, hematoma, thermal pain, nerve injury, and deep vein thrombosis (DVT) [8].

### 1.1. Study Objective

Given the widespread use of EVLA and its potential for complications, this study is aimed at systematically identifying risk factors associated with adverse outcomes. Recognizing these factors is essential for improving patient selection, ensuring procedural safety, and enhancing treatment effectiveness.

## 2. Patients and Methods

### 2.1. Study Design

This retrospective, observational study was conducted at a single tertiary care center between January 2020 and January 2024. It included a consecutive series of symptomatic patients diagnosed with primary VVs due to saphenous vein incompetence. The affected venous systems included the great saphenous vein (GSV), small saphenous vein (SSV), and/or accessory saphenous veins. Eligible participants had CEAP classifications ranging from C3 to C6 and presented with clinical signs and symptoms consistent with venous reflux. Our institutional pathway prioritizes conservative management for uncomplicated C2 disease (lifestyle measures and compression), reserving EVLA for C3–C6 or C2 patients who fail conservative therapy. During the study window, no C2 patients proceeded to EVLA; hence, no C2 data appear in the cohort. To avoid confusion, we have revised the methods to specify the analyzed cohort as consecutive CEAP C3–C6 patients undergoing EVLA. The study protocol received approval from the university's Institutional Review Board (IRB No. 357N/2025) and complied with the ethical standards outlined in the Declaration of Helsinki.

### 2.2. Preoperative Evaluation

All patients underwent a thorough preoperative workup, including a physical examination, duplex ultrasonography, CEAP staging, and assessment using the Venous Clinical Severity Score (VCSS). Duplex scanning was performed in the standing position to evaluate reflux, defined as reverse flow lasting longer than 0.5 s after manual calf compression. Reflux was assessed at multiple anatomical levels: the saphenofemoral junction, mid-thigh, lower thigh, and below the knee for the GSV and the saphenopopliteal junction, mid-calf, and lower calf for the SSV. Additional measurements included the skin-to-vein distance and the lowest point of reflux.

Patients with congenital vascular malformations, active DVT, postthrombotic syndrome, or nonsaphenous varicosities were excluded due to differing pathophysiology and expected treatment responses. However, patients with venous ulcers related to primary superficial insufficiency were included; those with ulcers caused by postthrombotic disease were excluded.

### 2.3. Treatment Protocol

Treatment strategies were guided by a standardized institutional protocol. Each patient received detailed counseling on the benefits and risks of all available interventions, including EVLA, and the final decision was made in collaboration with the patient. All procedures were performed by a single experienced vascular surgeon, following manufacturer guidelines without deviation from standard protocol.

Endovenous access was gained at the most distal site of reflux, with puncture performed below the saphenous fascia. Microphlebectomy was carried out simultaneously to treat residual varicosities. The choice between spinal and general anesthesia was based on patient preference and overall clinical status.

Postoperatively, all patients were advised to wear medical-grade compression stockings to reduce the risk of DVT. When tributary veins were located near the sural or saphenous nerves, microphlebectomy was performed under direct visualization—either with intraoperative ultrasound guidance or through extended incisions—to minimize the risk of nerve injury.

### 2.4. Postoperative Evaluation and Follow-Up

Follow-up assessments were scheduled at 2, 6, 12, 18, and 48 months. These included physical examination and duplex ultrasonography to evaluate vein patency, detect any thrombus, and assess for recanalization from the common femoral vein down to the popliteal vein. In patients with suspected junctional thrombus or symptomatic DVT, a full venous scan of the lower limbs was performed. In our cohort of 500 patients, 21 patients (4.2%) were lost to follow-up during the 48-month period. The reasons included patient relocation (*n* = 9), loss of contact (*n* = 7), and refusal to continue follow-up (*n* = 5). The overall attrition rate was therefore low. We performed the analysis on an intention-to-treat (ITT) basis, in which all enrolled patients were included in the statistical analysis up to their last available follow-up visit. Importantly, baseline demographic and clinical characteristics (age, sex, CEAP stage, comorbidities, and BMI) did not differ significantly between patients who completed the 48-month follow-up and those who were lost to follow-up, thereby minimizing the risk of attrition bias.

### 2.5. Outcomes

#### 2.5.1. Primary Outcome

The primary endpoint was the incidence of postoperative complications, including pulmonary embolism, DVT, myocardial infarction, transient recanalization, and recurrence of VVs.

Major complications were defined as death, pulmonary embolism, DVT, myocardial infarction, stroke or transient ischemic attack, visual disturbances, anaphylaxis, hemorrhage requiring transfusion or surgery, permanent neuralgia, or any event requiring hospital readmission.

Minor complications included wound infection, superficial thrombophlebitis, paresthesia, erythema, hyperpigmentation, lymphatic complications (e.g., lymphocele and edema), nonintervention hematoma, and hypersensitivity reactions.

#### 2.5.2. Secondary Outcome

Secondary outcomes included patient-reported improvements in quality of life, as well as procedural metrics such as operative time, access site, and stump length. Stump length was measured immediately postoperatively and again at 6 months. Recanalization was defined as the reopening of a previously occluded vein segment or a measurable increase in stump length over time.

### 2.6. Statistical Analysis

All data were analyzed using IBM SPSS Statistics, Version 21. Descriptive statistics (frequency, percentage, mean, and standard deviation) were used to summarize baseline characteristics and outcome data. Inferential statistics were conducted using the chi-square test, and differences in survival curves were assessed using the log-rank test. A *p* value <0.05 was considered statistically significant. Time to clinical recurrence was estimated using the Kaplan–Meier survival analysis. Procedural satisfaction was measured using follow-up VCSSs.

## 3. Results

**Figure 1:**
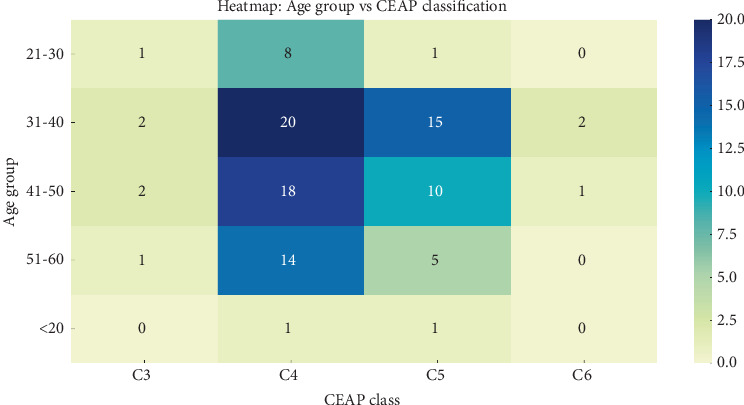
Heatmap showing the distribution of age groups against CEAP classification in patients with varicose veins.

A total of 500 patients underwent EVLA during the study period. The majority of the cohort were female (56.8%), while men constituted 43.2%. The mean age of the study population was 38 years (range, 16–60). The largest age group was 31–40 years (39%), followed by 41–50 years (31%), 21–30 years (21.6%), and 51–60 years (6.8%). Only 1.6% of patients were aged <20 years.  Figure 1 illustrates the distribution of CEAP classification across age groups, highlighting a concentration of C4 and C5 cases in the 31–50-year range.

All patients presented with tortuous varicosities, pain, and cosmetic deformities. Additional symptoms included leg edema in 1.6% of patients, venous ulcers in 2.6%, pigmentation changes in 1.2%, and telangiectasia in 0.3%.

A substantial majority of patients (89.2%) were either housewives or self-employed/free workers. Other occupations included barbers (4.0%), office employees (3.6%), restaurant workers (1.2%), and street vendors (0.8%). Of the 500 patients, 84 (16.8%) were current smokers, and the remaining 416 (83.2%) were nonsmokers.

**Figure 2:**
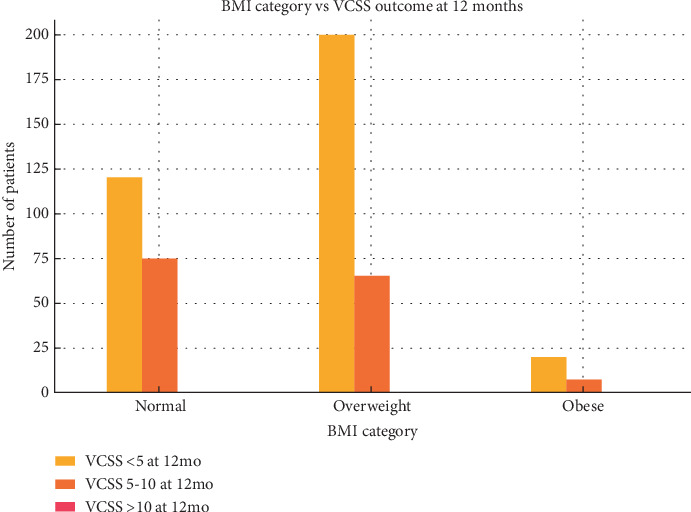
Distribution of BMI categories versus VCSS outcomes at 12 months of follow-up.

Over half of the patients (54.8%) were classified as overweight (BMI 25–29.9), while 39% had a normal BMI (18.5–24.9), and 6.2% were classified as obese (BMI > 30). As seen in Figure 2, patients with normal and overweight BMI had better VCSS outcomes at 12 months, with the majority achieving VCSS < 5. A smaller proportion of obese patients also showed improvement, although outcomes were less favorable overall.

**Figure 3:**
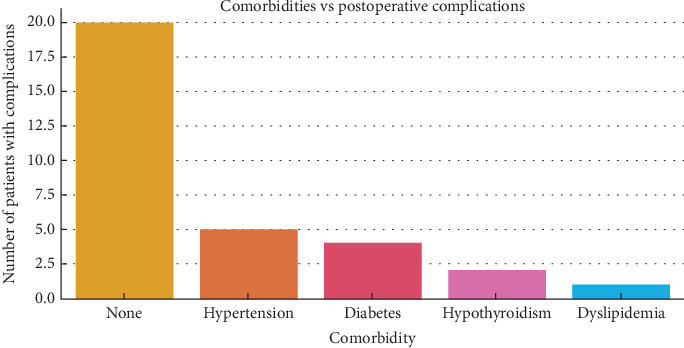
Relationship between patient comorbidities and postoperative complications.

Most patients (90.4%) had no known comorbidities. Among the remainder, 4.6% had hypertension, 2.6% had diabetes mellitus, 1.4% had hypothyroidism, and 1.0% had dyslipidemia. The relationship between comorbidities and postoperative complications is depicted in Figure 3. The majority of complications occurred in patients without comorbidities, likely reflecting their predominance in the cohort. However, hypertension and diabetes were most frequently associated with complications among the comorbid population, followed by hypothyroidism and dyslipidemia.

The majority of patients were classified as CEAP C4 (*n* = 309, 61.8%), followed by C5 (*n* = 162, 32.4%), C3 (*n* = 27, 5.4%), and C6 (*n* = 2, 0.4%). Preoperative Doppler ultrasound revealed that 82.6% (*n* = 413) exhibited Grade 3 venous reflux, 16.2% (*n* = 81) had Grade 4 reflux, and 1.2% (*n* = 6) had Grade 5 reflux (Table 1).

All patients received spinal anesthesia. EVLA was performed using a 1470-nm laser with an energy setting of 1490. Procedures included GSV leg and thigh ablation along with microphlebectomy. Accessory saphenous vein ablation was performed in 308 patients. All interventions were completed as day-case procedures.

Hematoma or ecchymosis occurred in 6.4%, swelling in 7.2%, and nerve injury in 3.6%. Nerve injury was assessed clinically during scheduled follow-up visits using both sensory and motor function evaluations. Sensory testing included pinprick, light touch, and temperature sensation along the distribution of the saphenous and sural nerves. Motor function was evaluated by grading ankle dorsiflexion and plantarflexion strength according to the Medical Research Council (MRC) scale. All identified cases represented transient sensory disturbances that resolved within 6–12 months; no permanent motor deficits were documented. A surgical site superficial infection occurred in 4% of patients. DVT occurred in 2% of patients, while the remaining 98% did not develop DVT. All cases of DVT were confirmed by duplex ultrasonography. The anatomical distribution was as follows: common femoral vein (*n* = 4), popliteal vein (*n* = 3), and posterior tibial vein (*n* = 3). No cases of iliac vein thrombosis were observed.

**Figure 4:**
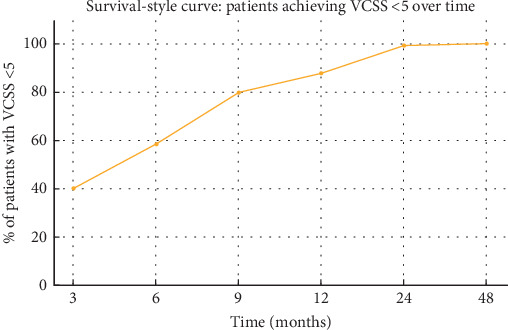
Survival-style curve showing the proportion of patients achieving VCSS < 5 over time.

Most patients (91%) exhibited no evidence of EHIT and were classified as “Not applicable.” EHIT was detected in a small proportion: Grade 1 in 3.8%, Grade 2 in 4%, and Grade 3 in 0.8%. There was a marked and progressive improvement in VCSSs during follow-up. At 3 months, 40.2% of patients had VCSSs < 5, 57.6% had scores between 5 and 10, and 2.2% had scores >10. At 6 months, 58.4% had VCSS < 5, increasing to 79.8% at 9 months and 87.8% at 12 months. By 24 months, 99.2% of patients had scores <5, and by 48 months, 100% achieved this threshold.  Figure 4 illustrates this improvement trend, with a survival-style curve showing cumulative patient improvement (VCSS < 5) over time (Table 2).

## 4. Discussion

This study provides meaningful insights into the risk factors and clinical outcomes associated with EVLA in a large, symptomatic patient cohort. Our findings confirm that EVLA is not only effective but also safe in the management of CVD, echoing international literature that has demonstrated its equivalence or superiority to traditional HL/S techniques [5–7].

Major complications were rare, with DVT occurring in just 2% of patients—a rate comparable to that reported by Chwała et al., who highlighted that strict procedural and postoperative protocols can significantly reduce thermal-related complications [8]. The use of spinal anesthesia in all cases, alongside routine compression therapy, likely played a role in reducing complication rates [9].

Minor complications such as hematoma (6.4%), swelling (7.2%), infection (4%), and nerve injury (3.6%) were consistent with rates observed in similar EVLA studies [10–12] (Table 3). Notably, nerve damage was infrequent, thanks to meticulous surgical technique and an emphasis on protecting the saphenous and sural nerve branches. There were no cases of pulmonary embolism or myocardial infarction, further underscoring the procedure's excellent safety profile.

Endothermal heat–induced thrombosis (EHIT) was also uncommon, affecting fewer than 9% of patients overall, with only 0.8% reaching Grade 3 severity. These outcomes support the value of postprocedural duplex surveillance in early EHIT detection and grading, as recommended in prior studies [13–16] (Table 4).

One of the study's most significant findings is the sustained clinical improvement demonstrated by the VCSS. By 48 months, all patients achieved a VCSS below 5, indicating durable symptomatic relief. This aligns with long-term data from Jiang et al. and Andercou et al., who reported superior quality-of-life outcomes following ETA when compared to conventional surgery [6, 7, 17, 18] (Table 5).

Our analysis also sheds light on the demographics and clinical profile of patients undergoing EVLA. The majority were female (56.8%), reflecting global trends that show a higher prevalence of VVs in women [19]. Most patients presented with CEAP Class C4 or C5 disease, suggesting that many seek medical attention at moderate to advanced stages. Despite this, high success rates were achieved, reinforcing the effectiveness of EVLA even in advanced cases.

BMI was another notable factor, with over half the cohort (54.8%) classified as overweight. While obesity is a known risk factor for CVD progression and postoperative complications [19–22], our findings suggest that favorable outcomes can still be achieved with appropriate perioperative planning. These results are in line with Costa et al., who emphasized that many risk factors for venous disease are modifiable and do not preclude successful intervention [3].

Interestingly, only 16.8% of patients were smokers, and overall comorbidity rates were low. While this could reflect regional patient characteristics or selection bias, it may also help explain the low complication rates observed [23–26].

## 5. Limitations

This study has several limitations worth noting. It is retrospective and single-center in design, which may limit the generalizability of the findings. Additionally, all procedures were performed by a single experienced vascular surgeon, which, while ensuring technical consistency, may not reflect the variability of practice in broader clinical settings. Nevertheless, the large sample size and extended follow-up duration contribute to the robustness and clinical relevance of our findings.

## 6. Conclusion

EVLA demonstrated excellent safety and efficacy in treating VVs in a diverse patient population. Major complications were infrequent, EHIT incidence was low, and long-term clinical outcomes were highly favorable, with 100% of patients achieving a VCSS below 5 by 48 months. Importantly, these results held true even in patients with elevated BMI and advanced CEAP stages. Collectively, our findings reinforce EVLA as a reliable, minimally invasive option for managing CVD. Future multicenter studies are recommended to validate these outcomes across broader patient populations and clinical settings.

## Figures and Tables

**Table 1:** Presentations, CEAP types, and reflux grades.

**Domain**	**Category**	**Frequency (** **n** **)**	**Percent (%)**
Clinical presentation	Tortuous veins, pain, disfigurement	469	93.8
	Leg edema	8	1.6
	Venous ulcer	14	2.6
	Pigmentation	6	1.2
	Telangiectasia	3	0.3

Initial CEAP score	C3	27	5.4
	C4	309	61.8
	C5	162	32.4
	C6	2	0.4

Doppler reflux grade	Grade 2	81	16.2
	Grade 3	413	82.6
	Grade 5	6	1.2

**Table 2:** Initial VCSS.

**Scores**	**Frequency**	**Percent (%)**
5–10	13	2.6
11–15	10	2
16–20	145	29
21–25	208	41.6
26–30	124	24.8

**Table 3:** Complications.

**Complications**	**No (frequency [percentage])**	**Yes (frequency [percentage])**
Hematoma/ecchymosis	468 (93.6%)	32 (6.4%)
Limb swelling	464 (92.8%)	36 (7.2%)
Nerve damage	482 (96.4%)	18 (3.6%)
Infection	480 (96%)	20 (4%)
DVT	490 (98%	10 (2%)

**Table 4:** EHIT grades and distributions.

**Grades**	**Frequency**	**Percent (%)**
Not applicable	455	91
Grade 1	19	3.8
Grade 2	20	4
Grade 3	4	0.8

Abbreviation: EHIT, endothermal heat–induced thrombosis.

**Table 5:** Postprocedure VCSS.

**Time (months)**	**V** **C** **S** **S** < 5**(%)**	**VCSS 5–10 (%)**	**V** **C** **S** **S** > 10**(%)**
3	40.2	57.6	2.2
6	58.4	30.4	1.2
9	79.8	19.6	0.6
12	87.8	10.2	2.0
24	99.2	0.8	0.0
48	100.0	0.0	0.0

Abbreviation: VCSS, Venous Clinical Severity Score.

## Data Availability

The data that support the findings of this study are available from the corresponding author A.B. upon request.
